# Does bariatric surgery improve ovarian stimulation characteristics, oocyte yield, or embryo quality?

**DOI:** 10.1186/s13048-014-0116-0

**Published:** 2014-12-10

**Authors:** Abraham Tsur, Raoul Orvieto, Jigal Haas, Alon Kedem, Ronit Machtinger

**Affiliations:** Infertility and In Vitro Fertilization Unit, Department of Obstetrics and Gynecology, Chaim Sheba Medical Center (Tel-Hashomer) and Sackler School of Medicine, Tel Aviv University, Tel Aviv, Israel

**Keywords:** Bariatric surgery, Morbid obesity, IVF, Female fertility

## Abstract

**Background:**

Obesity is a major global health concern associated with multiple co-morbidities. Bariatric surgery has been considered a good treatment option in cases of morbid obesity. This preliminary study aims to investigate the effect of bariatric surgery on ovarian stimulation characteristics and IVF treatment cycle outcome.

**Methods:**

A retrospective study that was performed in a tertiary, university-affiliated medical center and included all patients who underwent IVF treatment both before and after bariatric surgery. Data on ovarian stimulation variables of IVF treatment cycle prior and following the bariatric surgery were reviewed and compared.

**Results:**

From January 2005 to June 2014, seven women fulfilled the inclusion criteria. After the operation, BMI was significantly reduced (mean ± SD) (43.1 ± 3.3 vs. 29.6 ± 7.33, p = 0.018), as was the number of gonadotropin ampoules required during stimulation (69.3 ± 10.5 vs. 44.5 ± 17, p = 0.043). No between-cycle differences were observed in peak estradiol level, the number of oocytes retrieved, and percentage of mature oocytes.

**Conclusions:**

To the best of our knowledge, this preliminary case series is the first comparison of IVF cycle characteristics prior to and following bariatric surgery. The operation seems to reduce treatment costs without affecting oocyte or embryo quality. Further large studies are required to establish the surgery’s effect on IVF outcome among infertile women.

## Background

Obesity is a major global health concern with increasing prevalence [1]. According to the National Health and Nutrition Examination Survey (NHANES), the prevalence of obesity [body mass index (BMI) > 30 kg/m^2^] in the United States between 2011 and 2012 was as high as 36.1% among adult women, with a 8.3% prevalence of morbid obesity (BMI ≥ 40) [2]. Obesity may be associated with various co-morbidities such as diabetes type 2, chronic hypertension, heart disease, sleep apnea, and stroke [3]. Moreover, being obese may reduce woman’s fertility, increase risks associated with pregnancy [4], and be detrimental to in vitro fertilization (IVF) outcomes [5-8].

Treatment options include lifestyle modification, exercise, dietary and pharmacological therapy, liposuction, and bariatric surgery. While non-surgical approaches are generally associated with modest weight loss that is poorly sustained in most patients, bariatric surgery has been associated with significant weight loss, and its beneficial effects seem to be sustained in the long term [9].

Along the many merits of bariatric surgeries, these procedures might be followed by intraoperative, short and long term complications as wound infection, leakage through staples or sutures, abdominal hernia, ulcers, dumping syndrome, and nutritional deficiencies leading to anemia and osteoporosis [10].

Current indications for this operation are BMI ≥ 40 kg/m^2^ without co-morbidities or BMI ≥ 35 kg/m^2^ with severe co-morbidities [11,12]. Bariatric surgeries are classified as either mal-absorptive or restrictive [13] and are effective in reducing obesity-associated co-morbidities such as chronic hypertension and diabetes [13,14] and in improving spontaneous fecundity and pregnancy outcome [15]. However, data regarding the effects of bariatric surgery on the reproductive outcome of infertile women undergoing IVF are still scarce.

Prompted by the aforementioned observations, we aimed to investigate the effect of bariatric surgery on ovarian stimulation characteristics and IVF treatment cycle outcome in infertile patients undergoing IVF treatment, both prior to and following bariatric surgery.

## Methods

The study population consisted of all female patients admitted to our department between January 2005 and June 2014 with a history of bariatric surgery. We included only patients who underwent IVF treatment cycles both prior to and following bariatric surgery. The study was approved by the institutional IRB of Sheba Medical Center. Stimulation protocols employed for induction of follicular growth were previously described [16] and included long GnRH agonist protocol, flare GnRH-agonist protocol and the multidose GnRH antagonist protocol. Gonadotropines used included either recombinant FSH or HMG.

Data on patient age, BMI, and variables related to infertility treatment were collected from the files. Ovarian stimulation characteristics, number of oocytes retrieved, embryo quality, and number of embryos transferred during the patients’ IVF cycles before and after bariatric surgery were compared. Embryo classification was based on the individual scoring parameters according to pre-established definitions [17]. A top-quality embryo (TQE) was defined as seven or more blastomeres on day 3, equal sized blastomeres, and <20% fragmentation; all other embryos were categorized as poor quality.

Statistical analysis was performed using Wilcoxon rank sum test and Chi square, as appropriate p < 0.05 was considered significant.

## Results

Out of 9869 patients treated in our IVF unit during the study period, 18 had a history of bariatric surgery. Seven such patients underwent an IVF treatment cycle both before and after bariatric surgery. Five patients underwent sleeve gastrectomy and two gastric banding. Mean patient age ± SD at IVF cycles before and after the operation were 34.1 ± 5.69 years and 36.8 ± 6.1 years respectively. Reasons for IVF included male factor infertility (n = 2), PCOS (n = 2), genetic (n = 1), unexplained (n = 1) and age (n = 1). Mean ± SD intervals between the operation and the subsequent IVF treatment cycle and between the consecutive IVF cycles were 15.6 ± 7.9 months and 28.4 ± 29.0 months respectively. All the patients had their weight stabilized before starting the IVF treatment, as the metabolic parameters vary widely during the rapid weight-loss phase after the surgery. Of the seven patients, following the operation two had a normal BMI, two were overweight and three remained obese (of them two remained morbid obese).

The clinical characteristics of patients’ IVF cycles before and after the bariatric surgery are shown in Tables 1 and 2. BMI was significantly reduced after the operation (43.1 ± 3.3 vs. 29.6 ± 7.33, p = 0.018). Moreover, in two women severe co-morbidities (diabetes and hypertension) completely resolved following bariatric surgery without any additional treatment.

**Table 1:** **IVF treatment cycle characteristics of each patient undergoing IVF: last cycle before and first cycle after bariatric surgery**

**patient**	**Bariatric surgery**	**age**	**BMI**	**Total # of Gonadotropin ampoules**	**Days of stimulation**	**Peak E2 (pmol/L)**	**# of oocytes retrieved**	**# of MII oocytes**	**# of top quality embryos**	**# of embryos transferred**	**Type of Bariatric surgery**
# 1	Before	38	39.3	66	11	3025	6	3	0	2	Sleeve gastrectomy
	After	40	29.8	66	11	2562	4	4	0	4	
# 2	Before	36	42.7	50	11	4302	19	14	6	2	Sleeve gastrectomy
	After	42	25.6	40	11	4346	9	9	1	3	
# 3	Before	43	42	78	13	4167	2	2	1	2	Sleeve gastrectomy
	After	44	37.6	66	11	1065	1	1	0	1	
# 4	Before	26	47.8	71.5	25	1374	Retrieval cancelled – lack of ovarian response				Gastric banding
	After	27	38.9	30	10	4826	13	8	1	1	
# 5	Before	32	45.8	72	12	1158	7	5	1	4	Sleeve gastrectomy
	After	35	33	32	8	5444	10	8	3	3	
# 6	Before	29	39.1	*	*	*	15	10	*	2	Gastric banding
	After	31	20.7	*	*	*	20	16	3	4	
# 7	Before	35	45.2	78	19	4271	12	12	0	3	Sleeve gastrectomy
	After	39	21.5	33	13	4451.6	8	8	3	2	

*Data missing.

**Table 2:** **Summary of IVF treatment cycle characteristics of all patients undergoing IVF: last cycle before and first cycle after bariatric surgery**

	**Before surgery**	**After surgery**	**p**
**BMI**			
Mean ±SD	43.1±3.3	29.6±7.3	0.018
Median	42.7	29.8	
Range	39.1-47.8	20.7-38.9	
**Total number of gonadotropin ampoules used**			
Mean ±SD	69.3±10.5	44.5±17.0	0.043
Median	71.7	36.5	
Range	50-78	30-66	
**Days of stimulation**			
Mean ±SD	15.2±5.6	10.7±1.6	0.068
Median	12.5	11	
Range	11-25	8-13	
**Peak E**_**2**_ **(pmol/L)**			
Mean ±SD	3049.5±1462.6	3782.4±1642.5	0.46
Median	3596	4398.8	
Range	1158-4302	1065.0-5444.0	
**Number of follicles** **≥15 mm on day of hCG administration**			
Mean ±SD	3.8±2.3	7.0±4.2	0.1
Median	3	8	
Range	2-8	1-11	
**Number of oocytes retrieved**			
Mean ±SD	10.1±6.3	8.7±6.5	0.6
Median	9.5	9	
Range	2-19	1-20	
**Number of MII oocytes**			
Mean ±SD	7.7±5.0	7.7±5.0	1.0
Median	7.5	8	
Range	2-14	1-16	
**% MII of all oocytes retrieved**			
Mean ±SD	79%±21.3	90.9±11.5	0.1
Median	74	100	
Range	50-100	76-100	
**Fertilization rate** (%)	71.7%±21.5	67.9±26.5%	
Median	78.6	50	
Range	50-100	44-100	0.46
**Number of top-quality embryos**			
Mean ±SD	1.6±2.5	1.6±1.3	0.78
Median	1	1	
Range	0-6	0-3	
**% of top-quality embryos**			
Mean ±SD	28.2%±29.6	37.2±23.59	0.78
Median	25.0	42.8	
Range	0-66	0-75	
**Number of embryos transferred**			
Mean ±SD	2.5±0.8	2.8±1.1	0.51
Median	2	3	
Range	2-4	1-4	
**Number of embryos frozen**			
Mean ±SD	1.8±2.0	0.8±1.6	0.28
Median	1.5	1.5	
Range	0-4	0-4	

Following the surgery, patients required significantly fewer gonadotropin ampoules. There were no differences between the cycles in peak estradiol level, number of follicles ≥15 mm on day of hCG administration, the number of oocytes retrieved, MII oocytes, fertilization rates or number of TQE.

One of the patients conceived from embryos cryopreserved in the cycle following the operation. This pregnancy ended in missed abortion in the first trimester. Other patients did not conceive in the first cycle after the surgery.

## Discussion

In this preliminary case series we observed a significant decrease in the total number of gonadotropin ampoules required during IVF cycle following bariatric surgery, with no adverse effects on the number of follicles ≥15 mm, number of oocytes retrieved and number of MII oocytes.

To the best of our knowledge, this is the first comparison of IVF cycle characteristics prior to and following bariatric surgery. To date, few reports have described treatment outcomes of infertile female patients undergoing IVF following bariatric surgery. Doblado et al. published their description of five patients who underwent IVF treatment following bariatric surgery [18]. All their patients conceived in the first or second IVF cycle following the operation, and none experienced complications during or after IVF treatment cycles. The authors concluded that bariatric surgery is a safe procedure in women undergoing artificial reproduction techniques. Christofolini et al. compared ovarian stimulation parameters and treatment outcome among three different groups of patients: (a) 29 patients after bariatric surgery [average BMI 26.6 kg/m^2^, range (22.8-35.8)]; (b) 57 obese patients [average BMI 32.8 kg/m^2^, range (30.1-46.2)]; and (c) 94 normal-BMI and overweight patients [average BMI 23.5 kg/m^2^, range (17.5-29.6)] undergoing their first IVF cycle [19]. They reported significantly fewer follicles, oocytes retrieved, and mature oocytes among patients with a prior bariatric surgery as compared with patients from the two other groups. Although the difference was statistically significant, it seems clinically meaningless, since the mean number of follicles and oocytes retrieved were 5 and 5 (respectively) in the bariatric surgery group, 6 and 6 in the obese group, and 7 and 6.5 in the normal BMI/overweight group. However, their conclusions might deserve further consideration, as the difference might be attributable to the variability in ovarian reserve parameters among patients rather than the intervention.

The effect of bariatric surgery on female fertility and ovarian reserve is of specific significance, given the reported increase in the number of bariatric surgeries [20]. Therefore, evaluating the effects of this operation on female fertility has become quite relevant. Radical decreases in BMI after the operation have previously been described. The significant decrease in the number of FSH ampoules required after the surgery is associated both with reduced treatment costs and increased patient comfort, due to fewer injections.

Moreover, while the radical decrease in weight following surgery usually improves metabolic impairments such as diabetes, it might also cause deficiencies in iron, folate, vitamin B12, calcium, and vitamin D [21]. Animal and human data suggest that these nutrients might play a role in folliculogenesis and oocyte maturation [22-24], with deficiencies possibly causing detrimental effects on fertility.

Time to start treatment again was tailored by patient’s age, weight reduction and metabolic parameters. Possibly some of the beneficial effects of bariatric surgery on fertility were obscured by the time passed and the potential decrease in ovarian reserve due to aging. Historically women undergoing bariatric surgery were advised to delay pregnancy for 12 to 24 months after surgery. This recommendation was intended to both optimize weight loss and minimize adverse effects of nutritional deficiencies [25,26]. Recent studies and guidelines emphasize the importance of nutritional balance rather than the time from surgery to conception [27]. It might make sense to offer this treatment option to younger patients, seeking for infertility treatments, as in the older population the delay in time might interfere with fertility treatment results.

The main limitations of our study are the small sample size (seven patients) and the long period between the operation and the subsequent IVF treatments. In order to demonstrate, for example, a difference of 30% in the number of oocytes retrieved at a power of 80% and an alpha value of 5% using the uncorrected chi-squared test, 33 study subjects would be needed. This is a preliminary study and the sample size was limited by the unique methodology comparing IVF cycle characteristics in the same patients prior to and following bariatric surgery and the relative rarity of such patients.

## Conclusions

These preliminary case series suggest that bariatric surgery for weight reduction seems to be a safe and beneficial treatment option for morbidly obese infertile patients. Further studies are needed to evaluate the long-term effects of bariatric surgery on ovarian reserve or ovarian stimulation characteristics.

## Abbreviations

BMI: Body Mass Index
TQE: Top-Quality Embryo
